# The Role of Tumor and Host Microbiome on Immunotherapy Response in Urologic Cancers

**DOI:** 10.33696/cancerimmunol.6.078

**Published:** 2024

**Authors:** John Pfail, Jake Drobner, Krishna Doppalapudi, Biren Saraiya, Vignesh Packiam, Saum Ghodoussipour

**Affiliations:** 1 Section of Urologic Oncology, Rutgers Cancer Institute of New Jersey and Rutgers Robert Wood Johnson Medical School, New Brunswick, NJ, USA; 2 Department of Medicine, Division of Medical Oncology, Rutgers Cancer Institute of New Jersey, New Brunswick, NJ, USA

**Keywords:** Urologic oncology, Immunotherapy, Microbiome, Bladder cancer, Kidney cancer, Prostate cancer, Tumor microenvironment

## Abstract

**Introduction & Objective:**

The role of the microbiome in the development and treatment of genitourinary malignancies is just starting to be appreciated. Accumulating evidence suggests that the microbiome can modulate immunotherapy through signaling in the highly dynamic tumor microenvironment. Nevertheless, much is still unknown about the immuno-oncology-microbiome axis, especially in urologic oncology. The objective of this review is to synthesize our current understanding of the microbiome’s role in modulating and predicting immunotherapy response to genitourinary malignancies.

**Methods:**

A literature search for peer-reviewed publications about the microbiome and immunotherapy response in bladder, kidney, and prostate cancer was conducted. All research available in PubMed, Google Scholar, clinicaltrials.gov, and bioRxiv up to September 2023 was analyzed.

**Results:**

Significant differences in urinary microbiota composition have been found in patients with genitourinary cancers compared to healthy controls. Lactic acid-producing bacteria, such as *Bifidobacterium* and *Lactobacillus* genera, may have value in augmenting BCG responsiveness to bladder cancer. BCG may also be a dynamic regulator of PD-L1. Thus, the combination of BCG and immune checkpoint inhibitors may be an effective strategy for bladder cancer management. In advanced renal cell carcinoma, studies show that recent antibiotic administration negatively impacts survival outcomes in patients undergoing immunotherapy, while administration of CBM588, a live bacterial product, is associated with improved progression-free survival. Specific bacterial taxa, such as *Streptococcus salivarius*, have been linked with response to pembrolizumab in metastatic castrate-resistant prostate cancer. Fecal microbiota transplant has been shown to overcome resistance and reduce toxicity to immunotherapy; it is currently being investigated for both kidney and prostate cancers.

**Conclusions:**

Although the exact mechanism is unclear, several studies identify a symbiotic relationship between microbiota-centered interventions and immunotherapy efficacy. It is possible to improve immunotherapy responsiveness in genitourinary malignancies using the microbiome, but further research with more standardized methodology is warranted.

## Introduction

The microbes that inhabit our bodies— from the skin to the oral cavity and through the gastrointestinal and genitourinary tracts— exist in commensal relationships with our own cells. The collective assembly of bacteria, viruses, protozoa, and fungi in the human body forms a complex community, which is widely known as the human microbiome. Each site in the body harbors a unique microbial ecosystem that differs in both composition and metabolic function [1,2]. Over the last two decades, variations in the human microbiome have been extensively studied, revealing that the composition of an individual’s microbiome can be influenced by genetic and environmental factors such as diet, toxin exposure, and hormones [3,4]. Modern advancements in genomic sequencing technologies and metabolomics have led to a deeper understanding of the link between disruptions in the human microbiome and certain health conditions [5]. Dysbiosis, or persistent imbalances in the microbiome, has been linked to conditions like inflammatory bowel disease, obesity, and diabetes [6,7]. Moreover, dysbiosis is associated with a greater risk of immunopathology from deviations in cellular signaling, which may provoke carcinogenic processes [8–10]. Although the number of microorganisms known to directly cause cancer remains small, changes in the microbial signatures are complicit in cancer through their influence on the immuno-oncology-microbiome axis [11].

The role of the urinary microbiome in the development of genitourinary malignancies is just starting to be appreciated. Since urine was formerly considered sterile, the urinary microbiome has only been recently described using enhanced culture methods to characterize bladder bacteria in standard urine culture-negative samples [12,13]. Research has demonstrated the link between urinary dysbiosis and non-malignant pathologies like female urge incontinence, and postoperative urinary tract infection risk in women undergoing urogynecology procedures has been connected to preoperative depletion of *Lactobacillus iners* in the urinary microbiome [14–16]. Investigators have also explored the male urinary microbiome, finding that dysbiosis is associated with males experiencing lower urinary tract symptoms and chronic pelvic pain syndrome [17,18]. Given these findings, comparisons of the urinary microbiome in patients with and without various urologic malignancies were undertaken, discovering differences in microbial signatures that suggested the urinary microbiome influences genitourinary neoplastic processes [19,20]. Furthermore, distinct differences in the composition of the urinary microbiome across sexes have been identified, and it is suggested that this disparity in genitourinary flora may contribute to the increased incidence of genitourinary cancers in men [21].

The development of immune checkpoint inhibitors and their use in several malignancies, such as kidney and bladder cancer, has revolutionized the cancer treatment landscape. Unprecedented improvement in progression-free and overall survival, especially in patients with advanced disease, has highlighted that drugs targeting cytotoxic T-lymphocyte antigen (CTLA-4) and programmed death/ligand 1 (PD-1/PD-L1) are valuable for disrupting tumor growth and spread [22]. In fact, accumulating evidence suggests that the microbiome can modulate the efficacy and toxicity of immunotherapy by augmenting certain signals in the highly dynamic tumor microenvironment [23,24]. Several studies have shown that microbiota-centered interventions can improve response to immunotherapy in non-genitourinary cancers such as melanoma, hepatocellular carcinoma, and colorectal carcinoma [25–28]. Conversely, dysbiosis in the microbiome has been correlated with primary resistance to both PD-1/PD-L1 and CTLA-4 immunotherapies [29–31]. In a prospective trial of 69 advanced renal cell carcinoma patients, antibiotics altered gut microbial fingerprints and reduced objective response rates to nivolumab from 28% to 9% (p <0.03), indicating that optimal responses to immunotherapy require a healthy, commensal microbiome [32].

Ultimately, the evaluation of a patient’s microbiome and its function is critical for understanding how our therapies including immunotherapies will impact patient’s malignancy. While numerous immune checkpoint inhibitors are approved for the treatment of genitourinary cancers, predictive biomarkers for response are still lacking [33]. While contemporary research has confirmed the importance of the gut and urinary microbiomes in the pathogenesis of several genitourinary malignancies, robust evidence regarding the impact of the microbiome on responsiveness to immunotherapy for these malignancies is limited. A detailed examination of contemporary data that reports associations between specific microbial taxa and immunotherapy response in urologic oncology is needed. Thus, the objective of this review is to summarize our current understanding of the human microbiome’s role in modulating and predicting immunotherapy response to genitourinary cancers. In particular, this review will synthesize the most up-to-date evidence by highlighting microbial signatures associated with significant changes in immunotherapy response across bladder, kidney, and prostate cancer.

## Methods

We conducted a literature search for research studies and review articles related to the human microbiome, immunotherapy, and cancers of the genitourinary tract. Our search included peer-reviewed publications available through PubMed and Google Scholar as well as preprints available through bioRxiv from the earliest available publication date in each database up to September 2023. The advanced search feature was used to query each of the databases using specific search terms. Keywords in our search included microbiome, microbiota, tumor microenvironment, immunotherapy, immune-checkpoint inhibitors, PD-1/PD-L1, CTLA-4, bladder cancer, urothelial carcinoma, kidney cancer, renal cell carcinoma, prostate cancer, prostate adenocarcinoma, testicular cancer, germ cell cancer, seminoma, non-seminoma, penile cancer, and penile squamous cell carcinoma. Abstracts were excluded, but any English-language randomized controlled trial, meta-analysis, systematic review, prospective, or retrospective study that focused on the role of the microbiome in urologic cancer was included in our review. Publications that reported observational findings, such as those which characterized microbiome differences across patients with urologic malignancies and healthy controls, were included for the sake of comprehensiveness. Evidence on microbiota-centered interventions in animal models were also included given the paucity of published research in this space. We did not find reported evidence linking the microbiome to testicular cancer or penile cancer; therefore, neither malignancy is covered in this review.

### The Microbiome and Immunotherapy Response in Bladder Cancer

One explanation for the increased incidence and mortality of bladder cancer in the elderly is that age-associated perturbations in gut and urinary tract microbiota induce systemic immune dysregulation with increased risk for tumorigenesis [34]. This process, also known as”inflammaging”, suggests that an improved understanding of age-related alterations to the gut and urinary microbiomes could provide insight into bladder cancer risk, recurrence, and treatment strategies [35]. For example, the urinary microbiome may be critical to the maintenance of urothelial cell junctions and therefore protection against harmful compounds or pathogens [36]. As we age, microbial dysbiosis caused by repeated exposure to waste products filtered by the kidney and excreted through the bladder can release genotoxins or carcinogenic metabolites which can induce neoplastic changes. Moreover, the urinary microbiome itself may convert pro-carcinogenic metabolites into harmful chemicals in the urine via organic processes like conjugation or deconjugation [35]. Research on schistosomal infections also provides evidence that the urinary microbiome can mediate malignant transformation. Adebayo et al. found that distinct microbial patterns existed in the urine of healthy patients, those who had schistosomal infections but no pathology, and those with schistosomal-induced squamous cell carcinoma [37].

Additionally, several studies have identified differences in the urinary microbiome between patients with and without urothelial cancer as well as in patients with non-muscle-invasive versus those with muscle-invasive disease. Although limited by small sample sizes, research has reported significant differences in both alpha diversity (microbial diversity within a sample) and beta diversity (microbial diversity across samples) in urothelial cancer patients compared to healthy controls [38–41]. Notably, a higher relative abundance of *Actinobacteria* and *Proteobacteria* phyla was observed in the urine of patients with urothelial cancer, and a higher relative abundance of *Firmicutes* phyla was observed in the urine of controls [38,42]. At the genus level, a higher abundance of *Actinomyces* and *Brucella* were present in the urine of patients with urothelial cancer, while *Lactobacillus* were significantly more abundant in urine samples from healthy controls [38,42]. Moreover, the relative abundance of *Lactobacillus* was higher in patients who did not develop urothelial cancer recurrence after treatment [40].

Differences in urinary microbiome composition may also help to identify patients who are most likely to respond to intravesical Bacillus Calmette-Guerin (BCG). Unfortunately, more than 40% of patients with non-muscle-invasive bladder cancer (NMIBC) treated with BCG exhibit recurrence, and up to 30% of these patients progress to muscle-invasive disease within five years [20]. Why some bladder cancers respond to BCG while others do not is still nebulous, but the idea that changes in the tumor microenvironment and urinary microbiome contribute to therapeutic response and can serve as predictors of response is credible. In pre-treatment voided urine samples from a trial of 31 patients treated for NMIBC with intravesical BCG, Sweis et al. reported higher relative concentrations of *Proteobacteria* in patients who ultimately had tumor recurrence (p=0.035), whereas *Lactobacillales* were more abundant in patients without tumor recurrence (p=0.049) [43].

The proposed mechanism by which the urinary microbiome may affect the efficacy of BCG is related to promoting or competing with the binding of BCG to fibronectin, a protein necessary for the expression of BCG-induced antitumor activity. Fibronectin attachment protein has a highly-conserved region of amino acid sequences that facilitates the stable attachment of BCG to the bladder epithelium, and the absence of stable fibronectin binding is associated with lower antitumor activity of BCG [44,45]. Bacteria that bind fibronectin, such as *Lactobacillus*, may potentiate fibronectin stimulation and heighten BCG’s ability to generate an immune response, resulting in improved clinical outcomes Indeed, *Lactobacillus iners*, which binds to fibronectin more superiorly than any other *Lactobacillus* species because it is equipped with fibronectin-binding adhesins, is associated with an amplified BCG response through the upregulation of fibronectin the superficial bladder [46]. Hussein et al. also recently reported that among 11 NMIBC patients, *Serratia, Brochothrix, Negativicoccus, Escherichia-Shigella*, and *Pseudomonas* were significantly more abundant in patients who responded to BCG compared to those who did not [47]. Although this study is limited by the fact that the 16S rRNA gene sequencing technology utilized can only identify bacteria at the genus and not species level, it underscores that urinary microbiota can synergize with BCG to amplify the treatment response. This is consistent with other immunological studies demonstrating commensal and probiotic bacterial strains that exhibit the ability to attenuate mucosal inflammation [24,48]. The actively recruiting SILENT-EMPIRE trial (NCT05204199) plans to investigate signatures in urinary and gut microbial profiles of NMIBC patients as predictors for BCG therapy response [49].

A more thorough understanding of the urinary microbiome’s influence on BCG response has led to the investigation of several biomarkers for predicting immunotherapy response. Recent evidence suggests that lactic acid-producing bacteria may have therapeutic value in augmenting immunotherapy response in bladder cancer. The presence or addition of *Bifidobacterium* and *Lactobacillus* genera in the bladder has been shown to induce apoptosis and provide antitumor properties through immune-mediated mechanisms [50]. *Lactobacillus rhamnosus* and *Lactobacillus casei* have specifically demonstrated anti-proliferative effects on bladder cancer cell lines *in vitro* and *in vivo* mouse models; in one study, *Lactobacillus casei* was even more cytotoxic to bladder cancer cells than BCG because it directly induced necrosis [51,52]. The probiotic *Lactobacillus casei* Shirota strain, found in fermented milk products, was shown to inhibit bladder carcinogenesis and significantly decrease superficial recurrence in mice, and a case-control study in Japan found that habitual intake of this lactic acid-producing bacteria reduced bladder cancer risk in humans. The proposed mechanism through which lactic acid-producing bacteria enhance immunotherapy response is by increasing the local expression of interferon-gamma and tumor necrosis factor-alpha, ultimately inducing neutrophil infiltration and macrophage phagocytosis of bladder mucosa [53–55].

Biomarkers that encourage T-cell infiltration into cancer cells, generating the “T-cell inflamed tumor microenvironment”, have also been associated with improved outcomes to immune-checkpoint inhibitors across multiple cancer types, suggesting that the cell-mediated immune response is key to the anti-tumor activity of drugs like ipilimumab [56,57]. Indeed, T-cell-inflamed gene expression signatures have been correlated with response to immune checkpoint inhibitors in large-scale trials of bladder cancer [58]. Nevertheless, many patients with higher-than-median T cell-inflamed gene expression signatures do not respond to immune checkpoint inhibitors, indicating that certain gene expression signatures may assist in identifying potential pathways causing resistance to initial treatment rather than in predicting treatment efficacy. For example, in the phase 3 results of the IMvigor130 trial, a positive T cell-effector gene expression signature did not correlate with improved overall survival in patients treated with the PD-L1 inhibitor atezolizumab compared to those treated with platinum-based chemotherapy alone [59]. In contrast, high fibroblast TGF-β-response gene expression signatures were associated with inferior overall survival in two trials of metastatic bladder cancer— one in which patients were treated with atezolizumab and one in which patients were treated with pembrolizumab [59,60].

Moreover, the prognostic value of PD-L1 expression in bladder cancer has been limited by the fact that its association with clinical benefit in immunotherapy treatment has been inconclusive [61,62]. Some data reports that PD-L1 expression is related to better objective response rates to immune checkpoint inhibitors [63]. However, the CPS score, which combines immune cell and tumor cell PD-L1 expression status, was not associated with improved overall survival for patients treated with pembrolizumab compared to chemotherapy in KEYNOTE-361 [64]. Thus, a positive PD-L1 status is only an indication to use anti-PD-L1 monotherapy in bladder cancer patients who cannot receive cisplatin.

Given the growing number of trials examining immune checkpoint inhibitors in advanced bladder cancer and BCG-unresponsive disease, the role of urinary microbiome-centered interventions to modulate these systemic immunotherapies will become an increasing area of investigation for clinician scientists. Evidence already exists that microbiota can significantly influence the efficacy of cancer chemotherapies; Geller et al. found that intra-tumoral *Mycoplasma hyorhinis*, as well as certain species of *Proteobacteria*, metabolize and inactivate gemcitabine in mouse models [65]. The interplay between the microbiome and various cancer drugs has been well-demonstrated in genitourinary cancers, and new data is emerging with respect to the immunostimulatory effects of certain microbes on CTLA-4 and PD-1/PD-L1 blockade [21]. Vetizou et al. found that the antitumor effect of CTLA-4 inhibition depends on *Bacteroides fragilis;* tumors did not respond to CTLA-4 blockade in antibiotic-treated mice, yet the defect could be overcome by gavage with *Bacteroides fragilis* [30]. Sivan et al. found that oral administration of *Bifidobacterium* in mice augmented the efficacy of anti-PD-L1 therapy by enhancing CD8(+) T-cell priming in the tumor microenvironment when compared to controls [66]. Most recently, Mager et al isolated three bacterial species—*Bifidobacterium pseudolongum, Lactobacillus johnsonii*, and *Olsenella* species— that significantly enhanced the efficacy of immune checkpoint inhibitors in four mouse models of cancer. These bacteria enhanced immunotherapy response by producing the metabolite inosine, which activated antitumor T-cells [67].

Intravesical BCG itself is now being studied as a potential immunomodulator in combination with systemic immunotherapy. In a study by Wang et al., intravesical BCG treatment clearly upregulated PD-L1 expression on bladder cancer cells and increased tumor-infiltrating CD8(+) T-cell activity [68]. These results suggest that BCG is a dynamic regulator of PD-L1, and recent evidence has corroborated this relationship; Hashizume et al. found that bladder cancer tissue that recurred after BCG immunotherapy had significantly higher PD-L1 expression than normal epithelial tissue that regenerated. CD8(+) T cells were more infiltrated in the recurrent bladder cancer tissue compared to the regenerated normal tissue as well. Moreover, strong increases in the expression of granzyme B, interferon-gamma and tumor necrosis factor-alpha were found to be released by the tumor-infiltrated CD8(+) T cells after BCG therapy [69]. Thus, the proposed mechanism for the synergy of BCG and PD-L1 inhibitors is that BCG can activate the adaptive immune system to enhance the cytotoxic effect of CD8(+) cells while upregulating PD-L1. Therefore, the combination of BCG and immune checkpoint inhibitors may be an effective strategy for bladder cancer management, although the efficacy and safety of this combination has yet to be validated in a randomized controlled trial. The POTOMAC trial (NCT03528694) is assessing the efficacy and safety of BCG in combination with the anti-PD-L1 therapy, durvalumab, compared to BCG alone in NMIBC [70]. The KEYNOTE-676 trial (NCT03711032) is examining BCG in combination with the PD-1 inhibitor, pembrolizumab, compared to BCG alone in high-risk recurrent (Cohort A) or naïve (Cohort B) NMIBC patients as well [71]. Similarly, BladderGATE (NCT04134000) is assessing the efficacy of combined Atezolizumab and BCG in patients with high risk NMIBC [72]. However, BCG therapy has also been shown to downregulate HLA-I and induce an immune subversion process in a subset of bladder cancer patients, highlighting that the immunomodulatory effects of BCG are complex and highly individualized [73]. Because fecal microbiota transplant (FMT) has been proposed as a safe and feasible tool for overcoming immune checkpoint inhibitor resistance in melanoma patients, the concept is plausible for treating immunotherapy or BCG-resistant bladder tumors [26,74]. No clinical trials are investigating FMT in urothelial carcinoma yet, but there are three trials examining the ability of FMT to improve efficacy and reduce toxicity of immune checkpoint inhibitors in renal cell carcinoma and metastatic castration-resistant prostate cancer [75].

Ultimately, the urinary microbiome is likely one piece of a larger puzzle that contributes to the complex pathophysiology and management of bladder cancer. While its value in modulating the response to systemic and intravesical immunotherapies certainly exists, further investigation is warranted to illuminate the exact mechanisms. Nevertheless, synbiotic or probiotic capsules to augment the efficacy of BCG and anti-PD-1/PD-L1 therapy represent a promising area of study. Furthermore, with the rapidly evolving landscape of immunotherapies for genitourinary malignancies, a closer examination of specific urinary microbial signatures and biomarkers may enable clinicians to optimize immunotherapy regimens based on whether these bacterial taxa and their metabolites have been demonstrated to promote or inhibit response to that therapy.

### The Microbiome and Immunotherapy Response in Kidney Cancer

In addition to bladder cancer, recent research efforts have focused on the synergy between immunotherapy and microbiome modulation in patients with renal cell carcinoma (RCC). Pal et al. conducted an observational study to characterize the stool microbiome of metastatic RCC patients receiving checkpoint inhibitors by assessing changes in patients’ microbiome composition throughout their therapeutic course. In this study, they collected stool samples from 31 patients before initiation of either nivolumab alone (77%) or nivolumab and ipilimumab (23%) and at 1- and 3-month follow-up. Overall, 58% of patients experienced clinical benefit, and the authors found that greater microbial diversity was associated with clinical benefit. Certain species, such as *Bifidobacterium adolescentis* and *Barnesiella intestinihominis*, were associated with enhanced clinical benefit to checkpoint inhibitors [76]. Following these results, their group conducted a randomized study to investigate the effects of the live bacterial product CBM588 in patients with metastatic RCC receiving nivolumab and ipilimumab. This study included 29 patients who were randomized to receive nivolumab and ipilimumab with (n=19) or without (n=10) CBM588. CBM588 is a nonpathogenic strain of *Clostridium butyricum*, a butyrate-producing anaerobic spore-forming bacterium. CBM588’s production of short-chain fatty acids is believed to restore healthy microbiota by spurring bifidogenic shift, specifically through augmentation of interleukin-17A-producing T cells and CD4[+] cells in the colonic lamina propria [77]. Median progression-free survival was significantly prolonged in the nivolumab–ipilimumab plus CBM588 arm compared with the nivolumab–ipilimumab alone arm (12.7 versus 2.5 months, HR 0.15, 95% CI 0.05–0.47, p<0.001). Although there was no significant change in the relative abundance of *Bifidobacterium* species from baseline to week 12 associated with nivolumab–ipilimumab with or without CBM588, a subgroup analysis revealed that there was a significant increase in response to treatment in patients who received CBM588. Furthermore, there were significant increases in specific chemokines, including CCL2, CCL4, CXCL9, and CXCL10, in patients receiving CBM588, but not in control arm patients, highlighting a potential mechanism for the observed effect of adding CBM588 to immunotherapy [78].

Routy et al. further analyzed the influence of the microbiome in mediating response to immunotherapy by evaluating 67 patients enrolled in clinical trials for advanced RCC. Oncologic outcomes were compared between patients who were prescribed antibiotics (beta-lactams, fluoroquinolones, or macrolides) for any reason within two months before or one month after starting immunotherapy with nivolumab or atezolizumab. Fascinatingly, antibiotic therapy was associated with a significant decrease in progression-free survival (7.4 vs. 4.3 months, p=0.012). The authors also found an overrepresentation of various bacterial species such as *Akkermansia muciniphila* in patients with longer progression-free survival, suggesting an enrichment of this species might help mediate the treatment’s efficacy [29]. Although still nebulous, the mechanism by which commensal bacteria like *Akkermansia muciniphila* improve immunotherapy efficacy is suggested to be through an interleukin-12-dependent recruitment of CXCR3(+)CD4(+) T lymphocytes into tumor beds. Initiation of PD-L1 inhibitor therapy also elicits local and systemic recall Th1-immune responses against existing gut flora like *Akkermansia muciniphila* that ultimately improves cancer immunosurveillance [79]. Another hypothesis, proposed by Mager et al., states that the disruption of the gut barrier by immunotherapy allows translocation of inosine produced by *Akkermansia muciniphila* into systemic circulation, which thereby activates T cells via the adenosine A2a receptor [67]. Thus, when antibiotics transiently disrupt the microbiome, the homeostatic consortia of microbes that govern the cancer-immune set point cannot function synergistically with immune-checkpoint inhibitors, resulting in reduced treatment efficacy. Similar results were discovered in a larger analysis completed by Lalani et al. This cohort included 709 patients who received antibiotic treatment (beta-lactams, fluoroquinolones, macrolides, or tetracyclines) within 8 weeks before to 4 weeks after initiation of an immune checkpoint inhibitor. The authors discovered that patients with recent antibiotic use experienced a significantly lower objective response rate (19.3% vs 24.2%; p=0.005), shorter progression-free survival (aHR: 1.15; 95% CI 1.04–1.30; p=0.008), and worse overall survival (aHR 1.25; 95% CI 1.10–1.41; p<0.001) [80]. However, the reported association between antibiotic use and worse clinical outcomes in RCC patients treated with immunotherapy requires further investigation. Antibiotic use may interfere with supportive microbiome-cytokine interactions, but several confounding factors make it difficult to draw strong conclusions from these results. These retrospective studies did not control for patients’ concomitant medications, pre-existing comorbidities, or any environmental influences on the microbiome, such as patient diet. These studies also lacked granular information about the indication, dose, and duration of antimicrobial use. Lastly, subtle biochemical differences in mechanistic drug pathways and lack of standardization in the antibiotic-immunotherapy combinations leaves room for confounding, especially given the complexity of the immunologic relationship between the microbiome and cancer therapy.

In addition to recent antibiotic use, Derosa et al. demonstrated that the administration of tyrosine kinase inhibitors (TKI) can also influence the composition of the microbiome and impact the success of immunotherapy. Using whole genome sequencing and pairwise comparisons/fold ratio to identify bacterial fingerprints in stool samples, the authors studied 69 patients with advanced RCC before and after treatment with nivolumab. Similar to Pal et al., Routy et al., and Lalani et al., the authors found that recent antibiotic use (within 60 days of nivolumab) reduced response rates (28% to 9%, p<0.03). Additionally, Derosa et al. found that TKIs induced a significant shift in immunostimulatory commensals in the microbiome—suggesting that these microbes could be harnessed to improve the efficacy of immune checkpoint blockade in RCC patients [32].

Although the exact mechanism is hard to elucidate, these studies suggest a functional biologic relationship between the gut microbiome and immunotherapy efficacy. It is possible that there are innate immunogenic bacteria that are required for the activation of these cancer drugs, and that antibiotic administration results in their elimination [81]. In hopes of restoring these organisms and thereby improving responsiveness to immune checkpoint blockade, researchers have begun to focus on fecal microbiota transplantation (FMT) in mice. In their study, Routy et al. reported that FMT from patients with immunotherapy-responsive RCC to germ-free mice reproduced a successful anti-PD1 response in these mice [29]. Additionally, when these mice were exposed to antibiotic therapy, the anti-PD1 response was diminished.

Other ongoing clinical trials assessing the role of FMT include the PERFORM trial (NCT04163289), which is aimed at analyzing the effect of FMT on the occurrence of immune-related colitis associated with ipilimumab/nivolumab treatment [82]. The TACITO trial (NCT04758507) also aims to study the effect of FMT from patients who are immunotherapy-responsive on improving response to pembrolizumab plus axitinib in patients with advanced RCC [83]. The rationale for FMT as an emerging therapeutic approach in RCC stems from two seminal studies that showed FMT from immunotherapy-responsive melanoma patients enabled >30% of immunotherapy-refractive melanoma patients to overcome treatment resistance. FMT led to reprogramming of the recipient patients’ tumor microenvironment with increased CD8(+) T cell infiltration and interferon-gamma signaling as well as increased *Ruminococcaceae* and *Bifidobacteriaceae* species [26,74]. Additionally, in a recent multicenter phase I clinical trial of 20 melanoma patients, Routy et al. demonstrated that FMT from healthy donors is safe in the first-line setting in combination with immune checkpoint inhibitors [84]. These clinical findings support the investigation of microbiome-centered interventions like oral capsule FMT to overcome immune-checkpoint inhibitor resistance and improve immunotherapy efficacy without compromising safety in genitourinary cancers. However, key questions remain for future clinical trials that examine this approach in genitourinary malignancies, such as determining the most appropriate donor based on our existing knowledge of dysbiotic microbiome signatures, the timing and route of FMT relative to immunotherapy, and whether multiple FMTs are required. Questions still remain about the optimal bacterial compatibility between recipient and donor, and if the promising results seen in melanoma will be reproducible in other oncologic pathologies.

### The Microbiome and Immunotherapy Response in Prostate Cancer

Emerging studies have suggested that proinflammatory bacteria in the gut and urinary microbiome can influence prostatic inflammation and may contribute to carcinogenesis [85]. Shrestha et al. analyzed urine samples from men prior to prostate biopsy and then studied the urinary microbiome in biopsy-positive versus biopsy-negative patients. Interestingly, they identified a cluster of pro-inflammatory bacteria that was more abundant in the prostate cancer cohort than in healthy controls [86]. However, despite the anatomical location and physiological function of the prostate, few clinical trials have been conducted assessing the interplay between the genitourinary microbiome and immunotherapy response in patients with prostate cancer. Moreover, although immunotherapy has been minimally studied as a treatment for prostate cancer, given that there seems to be an association between the microbiome, the immune system, and cancer control, KEYNOTE-365 (NCT02861573) is examining pembrolizumab in various combinations with other immunomodulating therapies, such as steroids, for metastatic castration-resistant prostate cancer [87].

Since immunotherapy is not routinely used in prostate cancer, there is limited data on immunotherapy response in this malignancy relative to other genitourinary cancers. Rapid progress in researchers’ understanding of the tumor microenvironment has led some investigators to hypothesize that the reduced efficacy of immune checkpoint inhibitors in prostate cancer may be related to the human microbiome composition [88]. A recent study by Sfanos et al. aimed to assess differences in the gastrointestinal microbiome of healthy controls compared to men with varying clinical stages of prostate cancer. This study included 21 men with prostate cancer and found a greater abundance of species previously linked with responsiveness to anti-PD-1 immunotherapy, such as *Akkermansia muciniphila* and *Ruminococcaceae* species, in patients taking oral androgen deprivation therapy [89]. To further explore this possible relationship, Peiffer et al. performed 16S rRNA gene sequencing of fecal DNA from 23 individuals with metastatic castrate-resistant prostate cancer progressing on enzalutamide but just prior to treatment with pembrolizumab to determine whether certain features of the microbiome are associated with anti-PD-1 treatment response. Using multiple alpha and beta diversity metrics they found that global bacterial composition was similar between responders and non-responders [90]. However, certain bacterial taxa, such as *Streptococcus salivarius*, were consistently associated with response (defined as a >50% decrease in serum PSA or radiographic response). In fact, *Streptococcus salivarius* was the most differentially abundant species between responders and non-responders and was consistently elevated in responders across the sequencing results from multiple hypervariable regions in all three cohorts examined. *Streptococcus salivarius* is hypothesized to deliver probiotic activity through the production of lantibiotic bacteriocins and has been found to modulate PPAR-gamma expression of intestinal epithelial cells, indicating relevancy with respect to immunotherapy in prostate cancer [91,92]. Interestingly, *Akkermansia muciniphila* levels were reduced in responder samples, contrary to the previous findings reported by Routy et al. in renal cell carcinoma [29]. These conflicting findings suggest that the association between the microbiome and immune checkpoint inhibitor response may be unique to individual cancer pathology. Moreover, this inconsistency highlights that distinct microbes can have highly individualized functions, whether positive or negative, based on specialized immunologic interactions at the level of the tumor microenvironment or due to differences in the local microbiome composition across anatomic environments.

FMT has also been suggested as an option to improve response or overcome resistance to pembrolizumab as well as mitigate potential gastrointestinal side effects in prostate cancer patients [75]. One currently ongoing clinical trial (NCT04116775) is assessing the effect of FMT on immunotherapy response in patients with prostate cancer. In this trial, patients with biopsy-proven metastatic castration-resistant disease will undergo treatment with pembrolizumab for 4 cycles in addition to continued enzalutamide and androgen-deprivation therapy. Non-responders will then undergo FMT and be retreated with pembrolizumab for an additional 4 cycles. The primary outcome of this study is the percentage of participants with a PSA decline of ≥ 50% at any time point following FMT.

### Obstacles, Limitations, and Future Directions

Current evidence regarding the effect of the human microbiome on immunotherapy responsiveness for genitourinary malignancies is limited. It is challenging to isolate a causal relationship as each malignancy and its tumor microenvironment is incredibly complex and individualized. Furthermore, the synergy between the human microbiome and the treatment of genitourinary malignancies has only recently sparked the interest of researchers, so there is still significant variability in the microbiome sampling and reporting methods used across the studies being performed in this space. It is worth noting that the time and type of metagenomic sequencing used to analyze bacterial samples across the studies analyzed in this was inconsistent, and the statistical tools used to generate diversity indices and functional assays differed across studies. Some studies reported quantitative changes in microbial diversity, although alpha- and beta-diversity were not always strictly separated, while other studies reported functional differences in the microbiome. Non-standardized metagenomic methodology across these studies adds a layer of uncertainty with respect to interpreting these results cohesively and drawing broader conclusions about the implications of these results. Moving forward, we recommend that an expert panel reaches consensus on how to standardize microbiome research with respect to statistical analysis and reported metrics. Ultimately, it is important to acknowledge that the complex interplay between the microbiome and immunotherapy response is extremely idiosyncratic and therefore may be hard to completely standardize. Isolating a specific mechanism for the observed positive association between microbiota-centered interventions and immunotherapy response will likely always be confounded by inability to control for nutrition and other geographic or environmental factors that influence the composition and metabolic output of the microbiome.

Still, understanding the role of the microbiome can help with patient screening and further risk stratification in urologic malignancies.This is hindered by several procedural challenges, such as a lack of tools for properly collecting urine or fecal specimens from patients for microbiome studies. Moreover, it is difficult to isolate an anatomically distinct segment of the urinary system for microbial environment categorization from a routine urine sample. For example, a urine sample has microorganisms originating from all sites in the urinary tract—the urethra, bladder, kidneys, vagina, and prostate— all of which likely have highly-specific signatures that are important to characterize and may impact treatment in different ways. Ultimately, once a standardized collection or gold-standard “liquid biopsy” is defined, captured samples may be analyzed using gene sequencing, and a patient’s responsiveness to immunotherapy may be calculated, which will further guide clinicians for individualized patient counseling. This is similar to how recent advancements in bladder cancer technology have enabled the use of circulating tumor DNA (ctDNA) as a biomarker to both guide the use of and predict response to adjuvant immunotherapy [93].

The potential therapeutic value of continuing to research the microbiome and its effect on immunotherapy in urologic malignancy is becoming increasingly powerful and several clinical trials are currently investigating this topic (Table 1). If certain microorganisms are responsible for cancer initiation and/or progression, and if these microbes may additionally influence cancer treatment, modulating the microbiome may eventually provide a clear benefit to patient survival. Researchers have previously investigated the use of probiotic bacteria, such as the Shirota strain of *Lactobacillus casei* (among others), to reduce the recurrence of NMIBC [53,94]. These studies led to promising results, showing lower grades of disease in the probiotic-treated animals. Initial studies in humans showed that probiotics prevented secondary tumor growth and modulated cytokine production [95,96]. Although the sample sizes were small and there were high rates of discontinuation, probiotic administration was demonstrated to be safe and potentially effective for preventing the recurrence of superficial bladder cancer. Thus, as the body of evidence supporting a positive association between the microbiome and immunotherapy response in urologic malignancies grows, probiotics and other microbiota-centered interventions should continue to be investigated.

## Conclusion

Recent advancements in gene sequencing have allowed further research into the potential effect the microbiome has on the responsiveness of genitourinary malignancies to immunotherapy. As highlighted above, the microbiome likely plays a role in modulating responsiveness to immunotherapy in bladder, kidney, and prostate cancer. Nevertheless, there is still much to elucidate regarding the mechanistic interplay between the microbiome and each malignancy in terms of initiation, progression, and response to various treatments. Further multi-disciplinary research efforts and the results of ongoing randomized controlled trials are needed to successfully translate current findings in laboratory research to clinical decision-making in patient care.

## Figures and Tables

**Table 1.** Ongoing clinical trials assessing the microbiome in correlation with GU malignancies.

Clinical Trial	Bladder	Kidney	Prostate	Intervention	Outcome
NCT05037825 (PARADIGM)	-	Yes	-	ICI	Changes in microbiome from baseline and at the end of ICI cycle 2
NCT03383107	-	-	Yes	Radiotherapy	Changes in immune mediators and microbiome following radiotherapy
NCT04687709	-	-	Yes	ADT	Changes in fecal microbiome at 3 and 6 months
NCT06153849	Yes	-	-	BCG	Comparison of urinary microbiome between relapsed and non-relapsed patients
NCT04638049 (IMPRINT)	-	-	Yes	ADT + RT	Changes in intestinal microbiome
NCT04204434	-	Yes	-	ICI	Evaluate fecal microbiome changes correlated to response and toxicity
NCT04775355	-	-	Yes	ADT + RT vs RT alone	Changes in microbiome following RT
NCT04114136	Yes	Yes	-	ICI	Time to progression/recurrence correlated to oral and stool microbiome
NCT03819296	Yes	Yes	-	ICI	Comparison of stool microbiome correlated with AEs
NCT06126731 (PROMIZE)	-	-	Yes	Enzalutamide + antibiotics	Response rate of antibiotics combinations in patients with mCRPC
NCT05753839 (SEVURO-CN)	-	Yes	-	Cytoreductive nephrectomy + ICI	OS; secondary endpoints include OS and PFS correlated to fecal and urine microbiome
NCT03888742	-	-	Yes	ADT	Differences in fecal and urinary microbiome in patients treated with or without ADT
NCT04669860	-	Yes	-	Observational	Urine and fecal microbiome composition
NCT05354102	-	Yes	-	Nivolumab +/− BMC128	ORR, CR, and PR with combined BMC128 and nivolumab
NCT04243720 (IRIS)	-	Yes	-	ICI	Fecal microbiome changes associated with resistance to immunotherapy
NCT03087903	-	-	Yes	Grape Seed Extract	PSA trends correlated with fecal microbiome
NCT04402151 (PSMA SBRT-SIB)	-	-	Yes	SBRT	Changes in fecal microbiome
NCT05122546	-	Yes	-	Cabozantinib +/− Probiotics	Changes in fecal microbial diversity
NCT02234921 (DRibble)	-	-	Yes	Cyclophosphamide + DRibble and HPV vaccines	Microbiome changes correlated to response to treatment
NCT05487859	-	Yes	-	ICI + acarbose	Fecal microbiome changes associated with acarbose administration
NCT06044025	-	-	Yes	iADT + turmeric + metformin	Changes in fecal microbiome correlated with PSA relapse
NCT05850182 (ACTIDIET-PRO)	-	-	Yes	Lifestyle modifications	Changes in fecal microbiome associated with lifestyle changes
NCT05590624	-	-	Yes	Mediterranean Diet	Changes in fecal microbiome correlated with prostate tissue metabolomics
NCT04163289 (PERFORM)	-	Yes	-	Ipilimumab + Nivolumab +/− FMT	Occurrences of immune-related colitis; changes in fecal microbiome following FMT
NCT04090710 (CYTOSHRINK)	-	Yes	-	Ipilimumab + Nivolumab +/− SBRT	PFS; changes in fecal microbiome
NCT05726786 (INCyst Trial)	Yes	-	-	Immunonutrition	Microbiome changes predictive of postoperative complications
NCT04995809 (EPRIMM)	Yes	-	-	Radiotherapy	Changes in fecal microbiome associated with risk of GI toxicity
NCT06153849	Yes	-	-	BCG	Correlation between urinary microbiome and BCG efficacy
NCT04256616 (ICH-MIM-01)	Yes	-	-	Mitomycin C	Urinary microbiome composition correlating with MMC efficacy and staging/progression of disease
NCT03709485	-	-	Yes	Observational	Fecal microbiome associated with prostate cancer risk
NCT04107168	Yes	Yes	-	ICI	Fecal microbiome prediction of PFS
NCT04579978 (TIME)	Yes	Yes	-	ICI	Changes in fecal microbiome induced by ICIs
NCT05204199 (SILENTEMPIRE)	Yes	-	-	BCG	Fecal microbiome associated with BCG-responders
NCT04625556	Yes	Yes	Yes	Observational	Fecal and urine microbiome analysis with urologic malignancies
NCT03688347	Yes	Yes	-	ICI	Analysis of fecal microbiome after ICI treatment
NCT04758507 (TACITO)	-	Yes	-	FMT	Number of participants disease free
NCT04116775	-	-	Yes	FMT + Pembrolizumab + Enzalutamide	Anticancer effect of FMT
